# *In Vivo* Adaptation and Persistence of *Neisseria meningitidis* within the Nasopharyngeal Mucosa

**DOI:** 10.1371/journal.ppat.1003509

**Published:** 2013-07-25

**Authors:** Kay O. Johswich, Shannon E. McCaw, Epshita Islam, Anna Sintsova, Angel Gu, John E. Shively, Scott D. Gray-Owen

**Affiliations:** 1 Department of Molecular Genetics, University of Toronto, Toronto, Ontario, Canada; 2 Division of Immunology, Beckman Research Institute of the City of Hope, City of Hope National Medical Center, Duarte, California, United States of America; Northwestern University Feinberg School of Medicine, United States of America

## Abstract

*Neisseria meningitidis* (*Nme*) asymptomatically colonizes the human nasopharynx, yet can initiate rapidly-progressing sepsis and meningitis in rare instances. Understanding the meningococcal lifestyle within the nasopharyngeal mucosa, a phase of infection that is prerequisite for disease, has been hampered by the lack of animal models. Herein, we compare mice expressing the four different human carcinoembryonic antigen-related cell adhesion molecules (CEACAMs) that can bind the neisserial Opa protein adhesins, and find that expression of human CEACAM1 is necessary and sufficient to establish intranasal colonization. During infection, *in vivo* selection for phase variants expressing CEACAM1-specific Opa proteins occurs, allowing mucosal attachment and entry into the subepithelial space. Consistent with an essential role for Opa proteins in this process, Opa-deficient meningococci were unable to colonize the CEACAM1-humanized mice. While simple Opa-mediated attachment triggered an innate response regardless of meningococcal viability within the inoculum, persistence of viable Opa-expressing bacteria within the CEACAM1-humanized mice was required for a protective memory response to be achieved. Parenteral immunization with a capsule-based conjugate vaccine led to the accumulation of protective levels of *Nme*-specific IgG within the nasal mucus, yet the sterilizing immunity afforded by natural colonization was instead conferred by *Nme*-specific IgA without detectable IgG. Considered together, this study establishes that the availability of CEACAM1 helps define the exquisite host specificity of this human-restricted pathogen, displays a striking example of *in vivo s*election for the expression of desirable Opa variants, and provides a novel model in which to consider meningococcal infection and immunity within the nasopharyngeal mucosa.

## Author Summary

*Neisseria meningitidis* (*Nme*), a common cause of bacterial meningitis, are carried asymptomatically in the nasopharynx by a substantial proportion of healthy individuals. Their strict adaptation to the human as host has so far impeded the development of animal models to study the meningococcal lifestyle *in vivo*. While several human CEACAMs are recognized by the neisserial Opa protein adhesins, we show here that the expression of human CEACAM1 in transgenic mice is necessary and sufficient to allow nasal colonization by *Nme*. The dependence on human CEACAM1 is attributable to the Opa proteins, since intranasal infection with Opa-negative colonies of *Nme* selects for bacteria expressing Opa proteins, and genetically Opa-deficient meningococci are unable to colonize these animals. We use this new mouse model to examine how innate immune factors such as neutrophils and complement limit colonization. Furthermore, we compare how adaptive responses elicited by colonization and those generated by parenteral vaccination differentially confer sterilizing immunity. Together, this work provides the first evidence of the critical nature of Opa-CEACAM1 binding *in vivo*, demonstrates that this is a major determinant of the host restriction by *Nme*, and reveals a clear disparity between immune correlates of sterilizing immunity conferred by natural colonization versus parenteral immunization.

## Introduction

Invasive meningococcal disease is a suddenly striking, life-threatening condition with high mortality rates, even under intensive medical care [1]. Yet, despite its deadly potential, *Neisseria meningitidis* (*Nme*) is a normal resident of the healthy human throat [2]. The events that precipitate a transition from commensalism to invasive meningococcemia or meningitis remain a matter of contention that remain difficult to address in the absence of an experimental model. While several studies have described strategies to study *N. meningitidis* in the mouse [3], [4], [5], [6], [7], [8], the strict specificity of neisserial virulence factors precludes them from reproducing the intimate association of *Nme* with human mucosal tissues. Reflecting this host restriction, the neisserial colony opacity-associated protein adhesins (Opa) are integral outer membrane proteins that bind to select human carcinoembryonic antigen-related cell adhesion molecules (CEACAMs) [9], [10]. Numerous *in vitro* studies have observed Opa binding to CEACAM1, CEACAM3, CEACAM5 and/or CEACAM6, one or the other of which are expressed by most cells encountered by the meningococci [11], [12], [13], [14]. Opa binding to any one of these CEACAMs is sufficient to allow bacterial adhesion and, in the case of polarized epithelial cells, transcytosis across epithelial monolayers [15]. The cellular response upon Opa binding does, however, depend upon the receptor bound because each CEACAM has the potential to elicit a distinct signaling response [16], [17], [18], [19].

Here, we show that expression of a full-length human CEACAM1 transgene is necessary and sufficient to allow the meningococci to establish intimate attachment to the nasopharyngeal mucosa and prolonged colonization following intranasal challenge of mice. Mice expressing other human CEACAMs were not colonized, highlighting the central importance of CEACAM1 for meningococcal infection. In addition to providing the first evidence to support the critical contribution of Opa-CEACAM1 binding in the nasopharynx, this model reveals the *in vivo* phenotypic selection of expressed Opa variants that bind CEACAM1, and allowed us to detail the relative contribution of innate and adaptive immune processes that provide a barrier to meningococcal colonization and disease.

## Results

### *N. meningitidis* colonize the nasopharynx of *CEACAM1*-humanized mice

**Figure 1:**
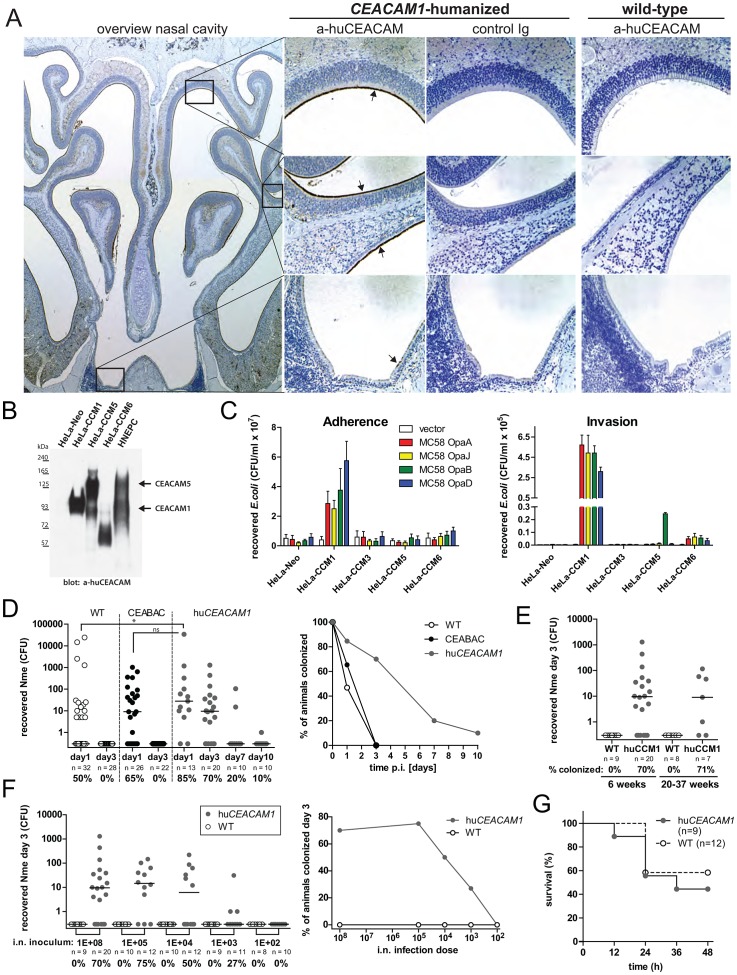
*CEACAM1*-humanized mice as model for *N. meningitidis* colonization. (A) Staining of mouse nasal sections for human CEACAM1. Overview of nasal cavity structures in low-magnification (5×) image indicates regions of interest for higher-resolution (20×) images. Human CEACAM1 is stained brown and indicated by arrows, nuclei appear in blue. Images are representative for *n* = 3 animals of each genotype. (B) Expression of CEACAMs analyzed in primary human nasal epithelial cells (HNEPC) in comparison to HeLa cells expressing defined CEACAM family members. Western blot was probed with rabbit polyclonal antibody (CEA-Dako) that recognizes each of these human CEACAMs. (C) Adherence to (left panel) and invasion into (middle panel) HeLa cells that express different CEACAM family members by *E. coli* expressing Opa proteins cloned from *Nme* strain MC58 assessed by gentamycin protection assay. Presented is the mean of *n* = 3 independent experiments ± SEM. Confirmation of Opa expression in *E. coli* is shown in Fig. S1C. (D) Cohorts of wild-type mice (open circles), CEABAC mice which express human CEACAMs 3, 5, 6, 7 (black circles) or *CEACAM1*-humanized mice (grey circles) were i.n. infected with 10^8^ CFU *Nme* strain MC58 and viable meningococci recovered from nasal tissues at indicated time points for quantitative culture. Data were pooled from at least two individual infections for each group. Horizontal bars indicate median. Kruskal-Wallis test applying Dunn's multiple comparison *post hoc* test was used to analyze the data, with * denoting *P*<0.05 and ns, not significant. Note that *CEACAM1*-humanized cohort at day 3 consists of pooled results from four independent experiments and is used as comparator in Fig. 1E, 1E, 5A, 5B and 5C. (E) Comparison of recovered meningococci from cohorts at day 3 after i.n. infection with 10^8^ MC58 at different ages. Note that 6-week old animal cohort is identical to Fig. 1D (day3). Horizontal bars indicate median. (F) Cohorts of mice were i.n. infected with varying CFU numbers of MC58 in the inoculum as indicated and viable bacteria recovered at day 3 post infection. Data were pooled from at least two individual infections for each group. Horizontal bars indicate median. Note that cohort of *CEACAM1*-humanized mice infected with 10^8^ CFU of MC58 are the same as in Fig. 1D (day 3). (G) Survival curves of wild-type or *CEACAM1*-humanized mice after intraperitoneal challenge with 10^6^ MC58.

CEACAM1 protein expression at the initial site of contact between *Nme* and the host nasopharyngeal mucosa is a prerequisite for this receptor to support meningococcal colonization. Consistent with its availability for Opa protein binding, the *CEACAM1*-humanized mice displayed human CEACAM1 protein on the apical side of the mucosa along the olfactory epithelium, the respiratory epithelium lining the maxillary sinuses and, in a spotted pattern, above the palate and the nasopharyngeal duct (Fig. 1A). The transgene is expressed under the control of the human *CEACAM1* promoter region, and careful histological analysis has established that its overall expression pattern matches well with that in humans [20], [21]. Reinforcing this important point, we confirmed that CEACAM1 and CEACAM5 are also expressed on primary human nasal epithelial cells (HNEPC) (Fig. 1B).

In order to define the binding potential of the prototypical serogroup B strain MC58 to the relevant human CEACAM family members, all four MC58 *opa* genes were expressed in *E. coli* and used for gentamycin protection assays. Each MC58 Opa protein was observed to mediate host cell binding and bacterial engulfment in a CEACAM1-dependent manner (Fig. 1C).

Next, we intranasally inoculated three cohorts of mice with *Nme*: *CEACAM1*-humanized mice, CEABAC mice (which express human CEACAM3, -5, -6, -7), and a control group of wild-type littermates. While meningococci could not be recovered from wild-type or CEABAC mice after the first day of infection, viable bacteria were recovered from *CEACAM1*-humanized mice for as long as seven days, with one transgenic animal still colonized at day ten (Fig. 1D). Carriage was not found to be age-dependent, since mice were still readily colonized at 5–7 months of age (Fig. 1E). Human CEACAM1 is, therefore, required for meningococcal colonization of the nasal tissues.

To further characterize this model, we determined the minimal dose required to successfully induce meningococcal carriage by infecting mouse cohorts with varying numbers of CFUs. As little as one thousand CFUs of *Nme* MC58 was sufficient to colonize one-quarter of the *CEACAM1*-humanized mice. With increasing CFUs in the inoculum, the carriage frequency increased such that 75% of the cohort was infected by 10^5^ CFUs (Fig. 1F).

The intranasally infected mice were routinely monitored for bacteremia, however none yielded a positive blood culture. To test whether human CEACAM1 impacts systemic infection by *Nme*, we used an intraperitoneal challenge model. Upon infection with 10^6^ CFU of *Nme* MC58, comparable survival curves were obtained with both genotypes (Fig. 1G). Therefore, human CEACAM1 drives nasal carriage, but does not seem to facilitate bacterial replication during disseminated infection in mice.

### *In vivo* selection of Opa^+^ phenotype during meningococcal colonization

**Figure 2:**
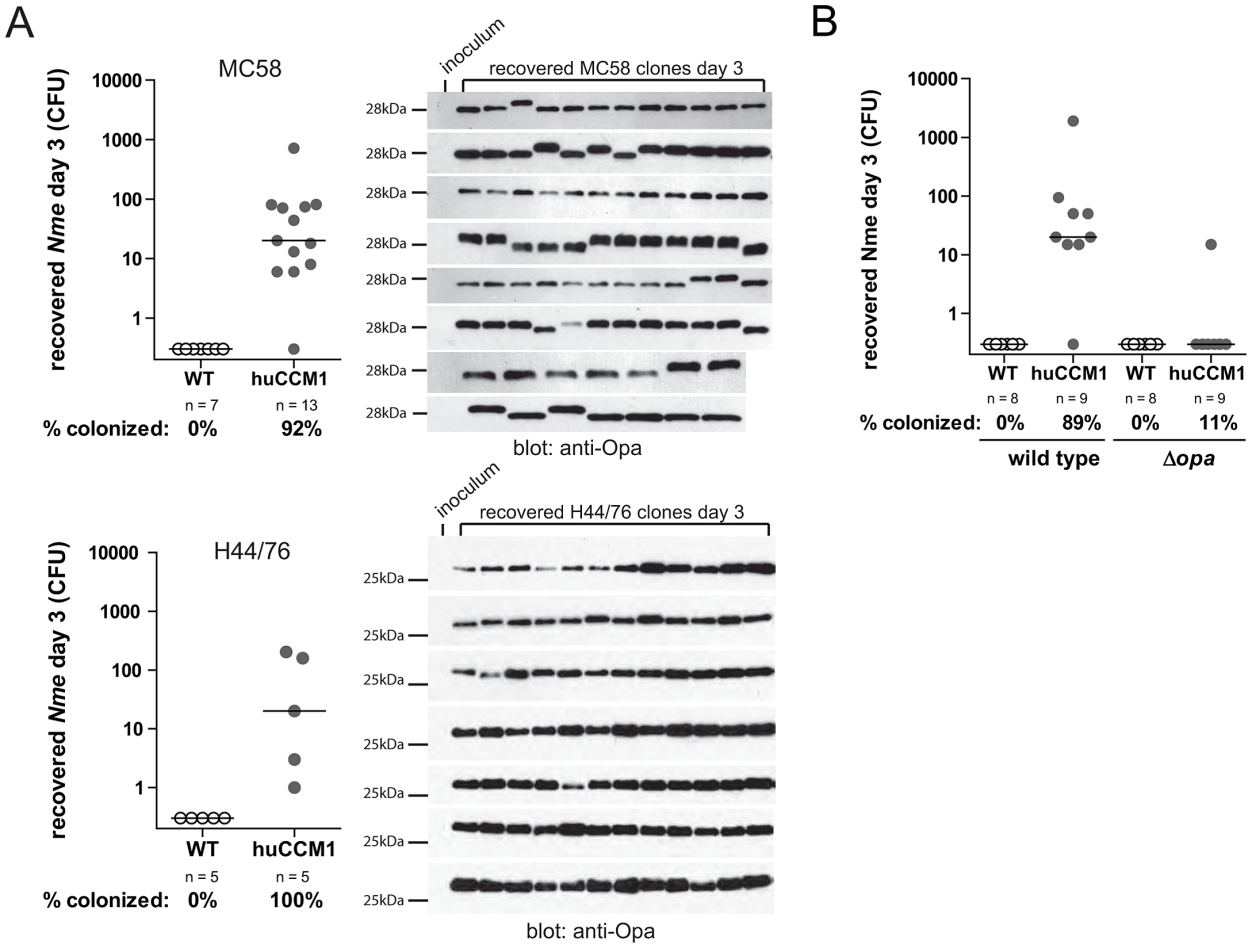
Opa/CEACAM dependency of meningococcal colonization. (A) Cohorts of mice were i.n. infected with 10^8^ CFU of serogroup B strains MC58 (upper panels) or H44/76 (lower panels) in which Opa protein expression was turned off by phase variation. Viable bacteria were recovered and enumerated at day 3 post infection (left panels). Data were pooled from two individual infections, horizontal bars indicate median. Western blot analysis was performed to monitor Opa expression in recovered clones (right panel). (B) Cohorts of mice were i.n. infected with 10^8^ CFU of strain H44/76 or an isogenic mutant (H44/76Δ*opa*) in which all four *opa* genes were disrupted. Viable bacteria were recovered and enumerated at day 3 post infection. Data were pooled from two individual infections for each group, horizontal bars indicate median.

Neisserial Opa protein expression is phase-variable, turning on and off randomly during bacterial cell division [22], [23]. When mice were inoculated with an *Nme* MC58 clone that had turned off Opa expression, *CEACAM1*-humanized mice were unexpectedly colonized at a rate comparable to mice infected with an Opa-expressing clone. However, strikingly, every recovered colony now expressed an Opa protein (Fig. 2A, upper panels; compare inoculum to recovered colonies), indicating that nasal colonization selected for an Opa^+^ phenotype. There was no obvious selection for any particular Opa variant(s) in vivo, presumably because all variants in this strain could bind CEACAM1 (Fig. 1C), making it likely that the frequency of expressing each allele would be related to the length of pentanucleotide sequences response for phase variable switching at each locus [24], [25]. This selection for Opa-expressing bacteria was replicated using an Opa^−^ clone selected from another prototypical serogroup B strain, H44/76 (Fig. 2A, lower panels), confirming that this was not a strain-specific response.

To confidently attribute the *in vivo* colonization to Opa-CEACAM1 binding, we disrupted all four *opa* alleles. Disruption of the Opa proteins did not cause gross defects in the strain, since their growth curve and protein expression pattern were indistinguishable from the parental strain (Fig. S1D and E). However, while the parental strain efficiently colonized the CEACAM1-humanized mice, the Opa-deficient bacteria almost entirely lost their ability to persist after intranasal infection (Fig. 2B). When combined, this represents the first direct evidence that the interaction between *Nme* Opa proteins and human CEACAM1 facilitates *Nme* colonization of the nasopharynx.

### CEACAM1-dependent attachment elicits an innate inflammatory response

**Figure 3:**
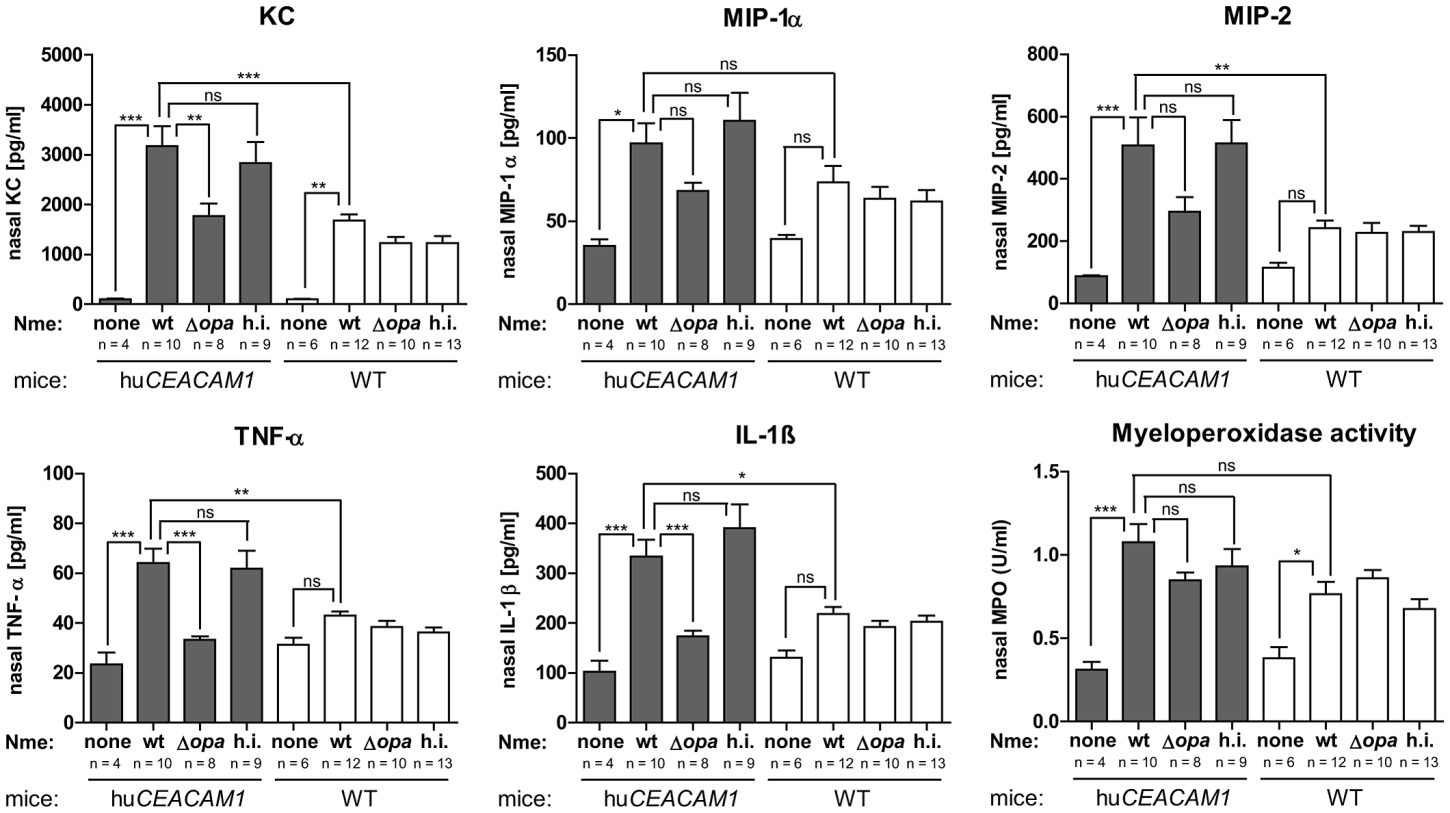
Inflammatory responses in nasal tissue after i.n. infection with *N. meningitidis*. Cohorts of mice were i.n. infected with 10^8^ CFU of H44/76wt (‘wt’), H44/76Δ*opa* (‘Δopa’), or heat-inactivated H44/76 (‘h.i.’), or left untreated (‘none’) and nasal tissues were harvested 16 h post infection to quantify chemokines KC, MIP-1α, MIP-1 and cytokines TNF-α and IL-1β by ELISA, or myeloperoxidase activity. Group sizes are indicated in graphs. *, **, or *** denote *P*<0.05, *P*<0.01, or *P*<0.001, respectively, in one-way ANOVA applying Tukey's *post-hoc* comparison of all groups using GraphPad Prism 5.0 software. ns, not significant.

*Nme* frequently live within the nasopharynx of healthy individuals, and are routinely considered normal flora when they remain at this site [26]. Considering that our model reflects this commensal state, we sought to assess whether asymptomatic colonization triggered an innate response. Upon infection with *Nme*, a robust inflammatory response marked by elevated chemokines KC, MIP-1α and MIP-2 as well as cytokines TNF-α and IL-1β was observed in the nasal tissue of *CEACAM1*-humanized mice and also, to a significantly lower extent, in wild-type mice (Fig. 3). While we are unaware of similar studies being performed in humans, these elevated cytokine responses reflect that seen in the nasal washes of children with *Haemophilus influenzae* [27], which is presumed to colonize a similar niche within the human nasopharynx. In the *CEACAM1*-humanized mice, heat-inactivated *Nme* caused an inflammatory response similar to that of the viable wild-type strain, whereas the cytokine response to viable but Opa-deficient meningococci was significantly lower; in the case of TNFα and IL-1β, the response to Opa-deficient meningococci was indistinguishable from the uninfected mice. Consistent with Opa-CEACAM1 binding being required for the augmented response, presumably due to more intimate mucosal association and/or prolonged persistence of the Opa-expressing bacteria, the wild-type mice responded to wild-type, Δ*opa* and heat-inactivated *Nme* in a manner reflecting that seen with Δ*opa* bacteria in *CEACAM1*-humanized mice.

### Polymorphonuclear cells suppress the meningococcal burden

**Figure 4:**
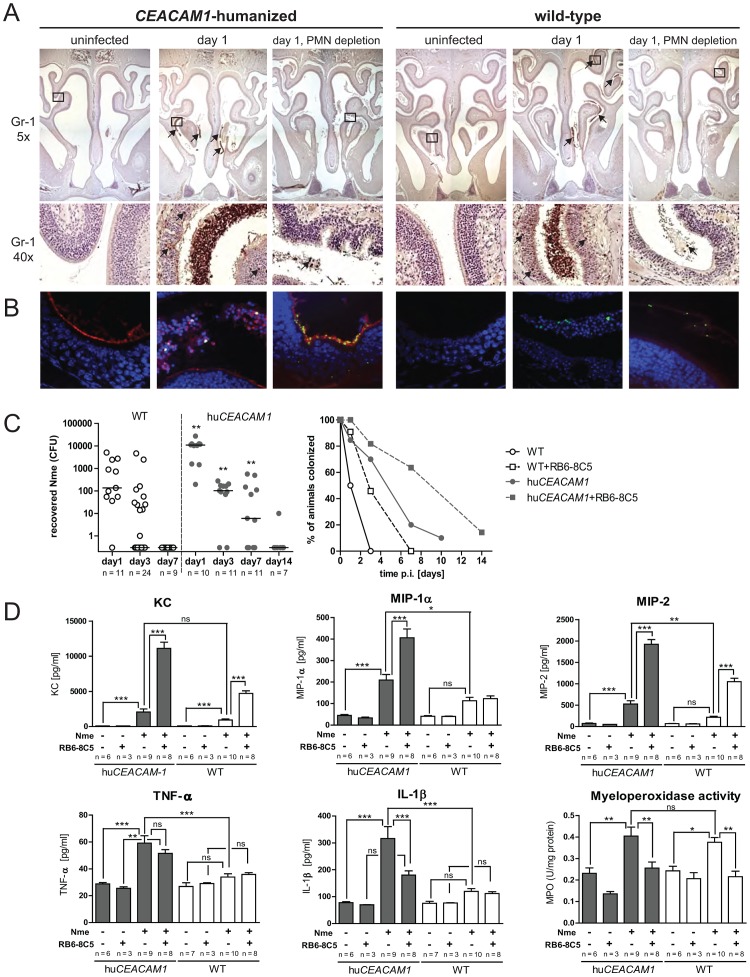
Role of polymorphonuclear cells in mucosal protection against *N. meningitidis* colonization. (A) *CEACAM1*-humanized and wild-tpye mice were either left untreated or were PMN-depleted by anti-Gr-1 antibody clone RB6-8C5 and then left either uninfected, or were i.n. infected with 10^8^ CFU MC58 as indicated. Immunohistological staining of nasal tissues for Gr-1 was performed on tissue samples obtained at day 1 to visualize PMN infiltrates. Overview of nasal cavity structures in low-magnification (5×) image on the top indicate regions of interest for higher-resolution (40×) images below. Gr-1 is stained brown and indicated by arrows, nuclei appear in blue. Images are representative for n = 3 animals of each genotype. (B) Same samples as in (A) were used for immunofluorescence staining. Human CEACAM appears in red, *Nme* in green and nuclei in blue. Note, that different regions of interest were chosen as compared to (A), but the images are in the same sequence as indicated for the groups above. (C) Cohorts of PMN depleted mice were i.n. infected with 10^8^ CFU of *Nme* MC58 and viable *Nme* recovered at indicated time points. Pairwise comparisons of PMN-depleted transgenic versus wild-type mouse cohorts were performed for each time point and analyzed using Mann-Whitney test; ** denotes *P*<0.01. Horizontal bars indicate median. Note that in the right panel plotting the percentage of colonized mice in each group, cohorts of PMN-sufficient mice from Fig. 1D were included for comparison. (D) Cohorts of untreated or PMN depleted mice were either left uninfected or were i.n. infected with 10^8^ CFU of *Nme* MC58 and nasal tissues harvested at 16 h post infection to quantify chemokines KC, MIP-1α, MIP-1 and cytokines TNF-α and IL-1β by ELISA, or myeloperoxidase activity. Group sizes are indicated in graphs. *, **, or *** denote *P*<0.05, *P*<0.01, or *P*<0.001, respectively, in one-way ANOVA applying Tukey's *post-hoc* comparison of all groups using GraphPad Prism 5.0 software. ns, not significant.

Myeloperoxidase, a marker for neutrophil infiltration, was found to be elevated in all inoculated mice (bottom right panel, Fig. 3), indicating involvement of PMNs in this model. Correspondingly, significant infiltrates of PMNs were found in the nasal mucosa and lumen of both *CEACAM1*-humanized and WT mice upon challenge (Fig. 4A). PMNs of both genotypes were positive for mouse Ceacam1, and in *CEACAM1*-humanized mice, they also expressed human CEACAM1 (Fig. S2A). To address the role of PMNs, we used an *in vivo* depletion strategy using the anti-Gr-1 antibody clone RB6-8C5 (see Fig. S2C and S2D).

In PMN-depleted mice, leukocytic infiltrates were virtually absent in nasal tissue (Fig. 4A). Interestingly, *Nme* infection was accompanied by increased damage of the mucosal tissues in *CEACAM1*-humanized mice depleted of PMNs, as lesions in the otherwise intact mucosal epithelium were visible (Fig. S2B); such damage was not apparent in animals where PMNs were not depleted. In accordance with a protective role for PMNs in phagocytic clearance of meningococci, the majority of meningococci in infected nasal tissues were associated with PMNs (Fig. 4B). Notably, *in vitro* infection experiments revealed that PMNs derived from bone marrow of *CEACAM1*-humanized mice bound meningococci more effectively than did those taken from WT mice (Fig. S3). When PMNs were depleted from the transgenic mice, *Nme* were more abundantly associated with the apical surface of the epithelium, corresponding with sites of human CEACAM1 expression, and in some instances even infiltrated into the tissue (Fig. 4B). Tellingly, the bacteria were restricted to the lumen and were not tissue-associated in the PMN-depleted wild-type mice.

Neutrophil depletion had a dramatic effect on *Nme* nasal colonization (Fig. 4C). In *CEACAM1*-humanized mice, the number of viable meningococci recovered from nasal tissues was much higher than in untreated mice (compared to Fig. 1C) at same time points. Moreover, viable bacteria could still be recovered from transgenic mice after 14 days under these conditions. Even in WT animals, PMN depletion lead to a transient susceptibility to meningococcal carriage. However, while viable *Nme* were not recovered from wild-type mice at day seven, 64% of the transgenic animals were still colonized at this point. Therefore, PMNs play a crucial role for the rapid clearance of meningococci within the mucosa, which partially masks the positive effect that human CEACAM1 has on *Nme* persistence within the tissues.

In addition to binding CEACAMs, Opa proteins can also facilitate inter-bacterial aggregation, which contributes to the colony opacity phenotype [28]. The transient susceptibility of PMN-depleted wild-type mice allowed us to assess whether *in vivo* selection for Opa protein expression depends upon the presence of human CEACAM1 (Fig. S4). While selection was not apparent in the wild type mice after one day infection (Fig. S4Bii, compare upper and lower panels) most colonies recovered after 3 days did express Opa. Together, these results suggest that mucosal CEACAM1 allows selective colonization by Opa-expressing *Nme* phase variants in the inoculum, but that Opa-dependent bacterial aggregation and/or other CEACAM1-independent benefits of Opa expression must also benefit *Nme* residing within the mucosa.

As expected, the increase in myeloperoxidase activity apparent during the response to infection was reversed when neutrophils were depleted (bottom right panel Fig. 4D). The nasal inflammatory cytokine response to infection was clearly altered upon PMN depletion. When neutrophils were absent, significantly higher levels of the chemokines KC, MIP-1α and MIP-2 were present when compared to neutrophil-sufficient *CEACAM1*-humanized mice, which, in turn, displayed strongly enhanced chemokine levels compared to non-infected mice (top panels Fig. 4D). In WT mice, a similar yet less pronounced trend was observed for the CXC chemokines KC and MIP-2 but not for the CC chemokine MIP-1α. Interestingly, the pro-inflammatory cytokines TNF- α and IL-1β showed a fundamentally different pattern than the chemokines (bottom left and middle panel Fig. 4D). TNF-α and IL-1β were both upregulated during infection of normal *CEACAM1*-humanized but not significantly in WT mice. Yet, upon PMN-depletion, TNF-α remained unchanged whereas IL-1β was actually decreased. Therefore, PMNs seemingly do not influence TNF-α but either directly or indirectly influence the release of IL-1β; whereas, in the absence of neutrophils, the increased bacterial burden and tissue damage leads to an enhanced chemokine response.

### Role of complement in nasal colonization of *N. meningitidis*

**Figure 5:**
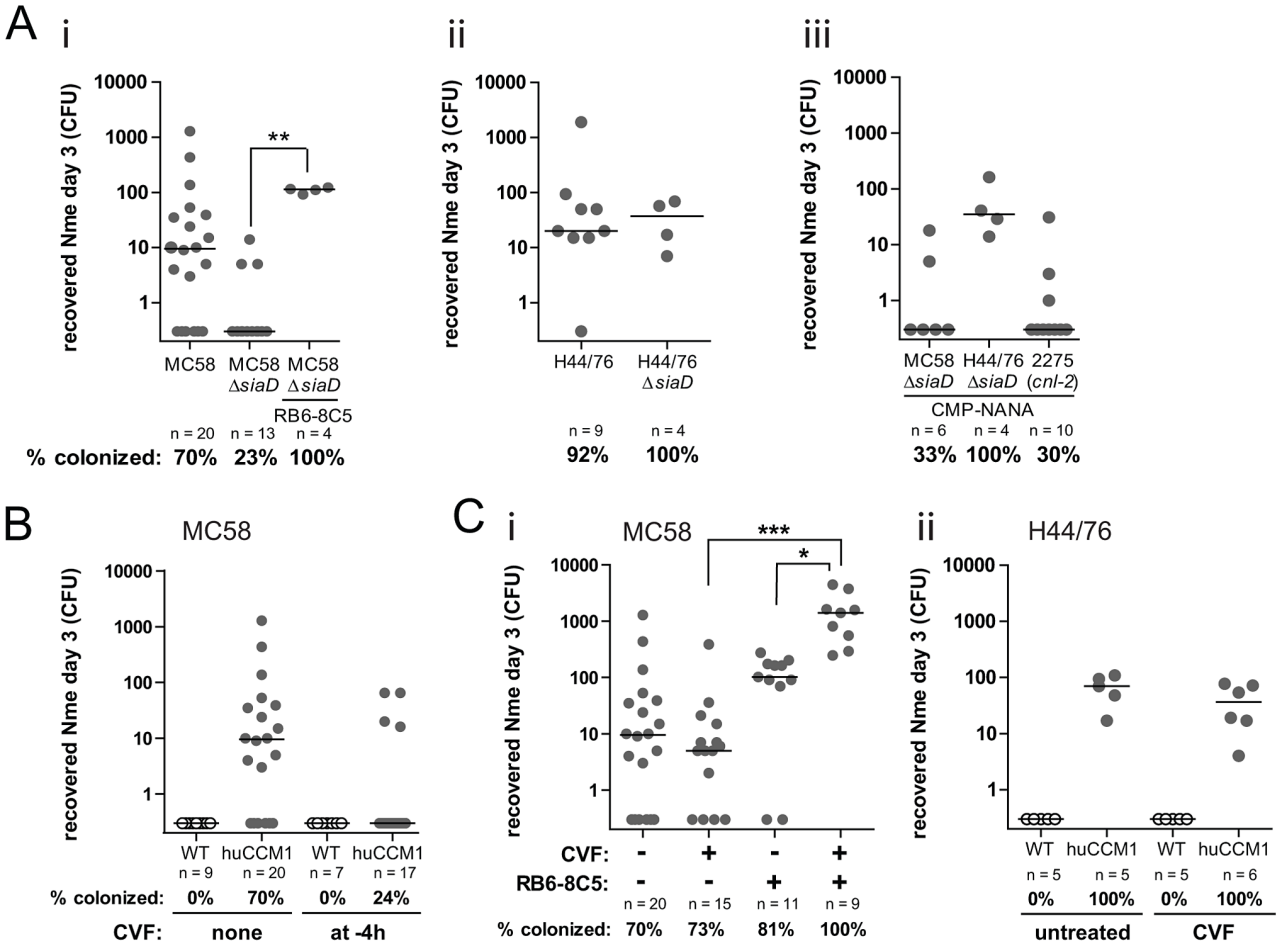
Role of complement in mucosal protection against *N. meningitidis*. (A): (i): Cohorts of mice were either left untreated or PMN depleted using the antibody clone RB6-8C5 and were subsequently i.n. infected with inoculi of 10^8^ CFU MC58 or MC58Δ*siaD* (capsule-deficient mutant). Viable bacteria were recovered and enumerated at day 3 post infection. Bars in graph denote median. * denotes *P*<0.05, using the Mann-Whitney nonparametric test. *CEACAM1*-humanized cohort infected with MC58 (encapsulated) is the same as in Fig. 1D (day3) and is shown here for comparison (not included in statistical test). (ii): Cohorts of mice were i.n. infected with 10^8^ CFU H44/76 or H44/76Δ*siaD* (capsule-deficient mutant) and viable bacteria recovered at day 3 post infection. Bars in graph denote median. (iii): Cohorts of mice were i.n. infected with 10^8^ CFU of MC58Δ*siaD*, H44/76Δ*siaD* or 2275, which is naturally non-capsulate because it harbors the capsule null locus 2 (*cnl-2*), grown in presence of 20 µM cytidine-5′-monophospho-N-acetylneuraminic acid (CMP-NANA). Viable bacteria were recovered and enumerated at day 3 post infection. Horizontal bars in graph represent median. (B) Cohorts of mice were i.n. infected with 10^8^ CFU of MC58 with or without receiving Cobra venom factor (CVF) 4 h prior to infection. Viable bacteria were recovered and enumerated at day 3 post infection. Data were pooled from at least two individual infections for each group, horizontal bars indicate median. Note that untreated control group is the same as in Fig. 1D (day3). (C): (i): *CEACAM1*-humanized mice were left untreated or received CVF alone, or anti-Gr-1 antibody clone RB6-8C5 alone, or both in combination at 30 h prior to infection with 10^8^ CFU of MC58. Viable bacteria were recovered and enumerated at day 3 post infection. Data were pooled from at least two individual infections for each group, horizontal bars indicate median. Significance levels are *, **, or *** denoting *P*<0.05, *P*<0.01, or *P*<0.001, respectively, using Kruskal-Wallis test applying Dunn's multiple comparison *post hoc* test. Note that untreated control group is the same as in Fig. 1D (day 3) and shown here to facilitate comparison. (ii): Cohorts of WT or CEACAM1-humanized mice were injected with CVF or left untreated at 24 h prior to i.n. infection with 10^8^ CFU of strain H44/76. Viable bacteria were recovered at day 3 post infection, horizontal bars in graph represent median.

The polysaccharide capsule of *Nme* is the major virulence factor protecting these bacteria against complement-mediated lysis and phagocytosis. Despite its importance during invasive disease, the capsule is thought to be dispensable for their ability to colonize the human nasopharynx [29]. Furthermore, *in vitro* studies suggest that the capsule sterically hinders adhesion to epithelial cells [30], [31]. In considering these points, we tested the impact of the capsule on colonization by comparing wild-type meningococcal strains MC58 and H44/76 with their corresponding isogenic capsule-deficient mutants, MC58Δ*siaD* and H44/76Δ*siaD*, respectively. To our surprise, MC58Δ*siaD* showed a decreased, yet not fully abrogated, ability to colonize the *CEACAM1*-humanized mice (Fig. 5Ai), whereas H44/76Δ*siaD* colonized the mice as well as the parental H44/76 strain (Fig. 5Aii), indicating strain-specific differences in the effect of capsule on colonization. Neutrophil-depletion allowed significantly increased colonization by MC58Δ*siaD*, suggesting that capsule confers resistance against phagocytosis or other neutrophil-mediated bactericidal mechanisms within the mucosa (Fig. 5Ai).

While most meningococcal disease is caused by encapsulated strains, invasive capsule-deficient *Nme* have recently emerged [32], [33], leading to suggestion that they have acquired novel virulence capacity. *Nme* strain 2275 is an invasive isolate that is naturally non-encapsulated and cannot produce CMP-NANA since it possesses the capsule null locus 2 (*cnl-2*) in place of the capsule operon. Despite this defect, strain 2275 was virulent in a mouse invasive challenge [33]. Perhaps related to this point, these strains can use exogenous cytidine-5′-monophospho-N-acetylneuraminic acid (CMP-NANA) as a substrate to decorate their lipo-oligosaccharide (LOS) with sialic acid so as to reduce complement deposition [34] and, in some strains, also impede nonopsonic phagocytosis [35]. Notably, strain 2275 colonized the *CEACAM1*-humanized mice to an extent comparable to the unencapsulated MC58Δ*siaD* (Fig. 5Aiii), suggesting that its increased virulence is not due to correspondingly increased fitness within the mucosal tissues.

Next, we sought to further explore the role of complement in controlling meningococcal nasal colonization. By injecting mice with cobra venom factor (CVF), an agent that elicits an unbridled complement cascade, we triggered massive complement activation shortly before intranasal administration of wild-type (encapsulated) *Nme* MC58. This treatment resulted in a significant reduction in colonization, indicating that activated complement components actually interfere with mucosal-associated meningococci (Fig. 5B).

Ultimately, CVF treatment leads to *in vivo* decomplementation lasting for several days, during which complement components are completely consumed (Fig. S5A and S5B). When mice were infected 30 h after CVF injection, by which time they were completely decomplemented, we surprisingly saw no effect on colonization with MC58 (Fig. 5Ci) or H44/76 (Fig. 5Cii). Therefore, while activated complement can combat infection within the mucosa (Fig. 5B), complement components do not normally play a critical role in controlling nasal colonization. However, when both complement and PMNs were depleted, a strong increase in bacterial burden was observed that was significantly greater than either of the treatments alone (Fig. 5C). Complement and neutrophils thus work synergistically as an innate barrier to counteract early-phase colonization.

### Persistent colonization is required to elicit sterilizing immunity

**Figure 6:**
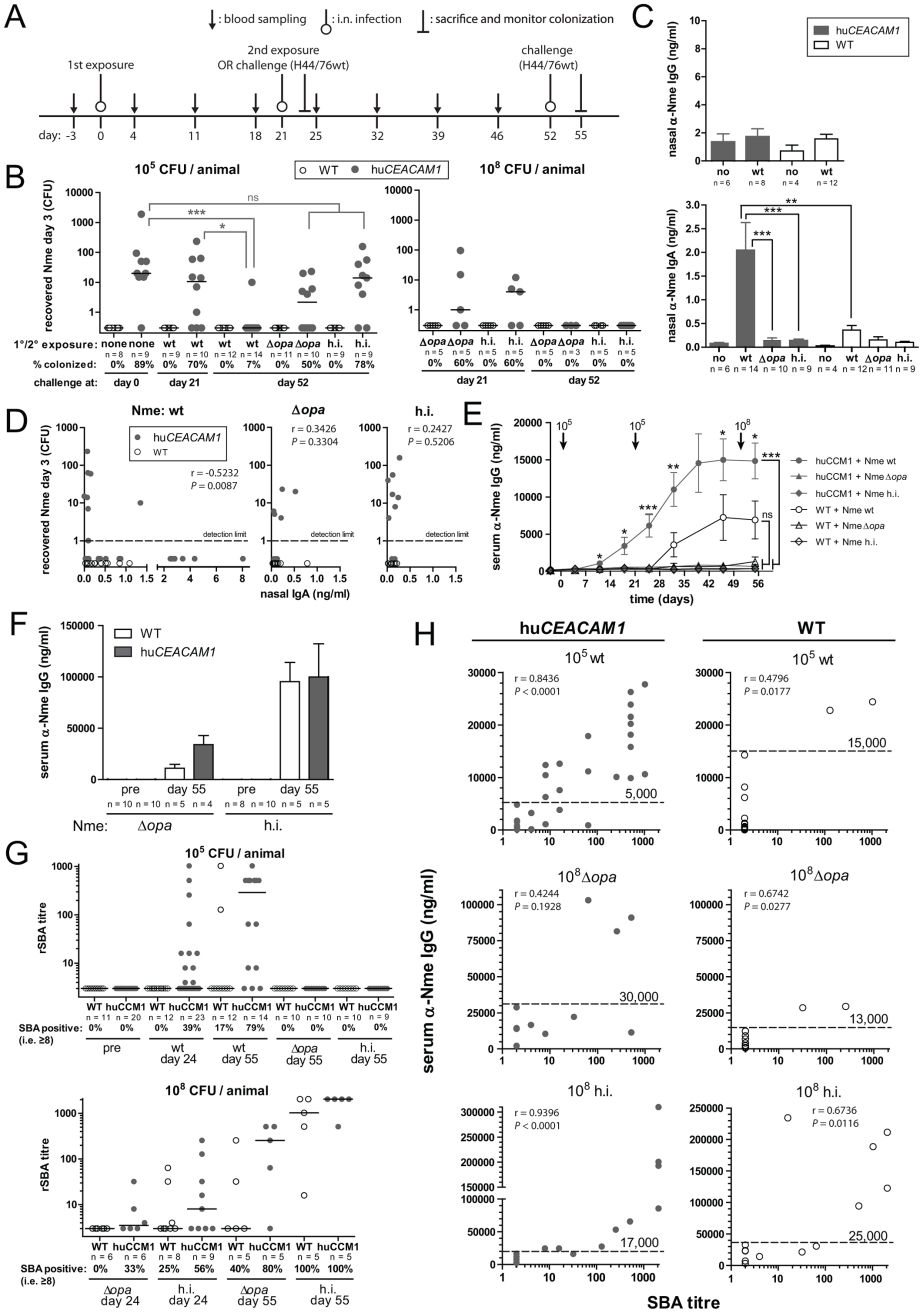
Generation of protective adaptive mucosal immune responses towards *N. meningitidis* after intranasal colonization. (A) Schematic representation of experiment. Cohorts of mice were i.n. infected with 10^5^ CFU of *Nme* H44/76 or either 10^5^ or 10^8^ CFU of H44/76Δ*opa* or heat-inactivated H44/76 at day 0 (first exposure) and again at day 21 (second exposure). Intranasal challenge infections with 10^8^ CFU of H44/76 were performed for some cohorts at day 0 or day 21, for all other cohorts at day 52 and mice were sacrifized after 3 days to recover and enumerate viable bacteria. (B) Resulting recovered viable meningococci after exposure to indicated inoculi of either 10^5^ (left panel) or 10^8^ (right panel), with ‘none’ = no previous exposure, ‘wt’ = H44/76wt, ‘Δopa’ = H44/76Δ*opa*, ‘h.i.’ = heat-inactivated H44/76 as indicated below x-axis. *, **, or *** denote *P*<0.05, *P*<0.01, or *P*<0.001, respectively, in Kruskal-Wallis nonparametric analysis applying Dunn's *post hoc* test. (C) Nasal lavage fluid was obtained from mice in (B) at day 55 and meningoccoccal (H44/76) specific IgG (upper panel) and IgA (lower panel) was determined by ELISA. Represented are mean values for each group, with group sizes indicated in graph. *, **, or *** denote *P*<0.05, *P*<0.01, or *P*<0.001, respectively, in one-way ANOVA applying Tukey's *post-hoc* comparison of all groups using GraphPad Prism 5.0 software. (D) Correlation of recovered CFU as in (B) with anti-meningococcal IgA concentrations in nasal lavage fluid as in (C) for each individual mouse exposed twice to 10^5^ CFU of the indicated inoculi before challenge, analyzed by Spearman's rank correlation test. (E) Meningococcal (H44/76) specific serum IgG concentration determined by ELISA in indicated treatment groups of experiment with 10^5^ CFU of the indicated inoculi used for exposures. Represented is the mean ± SEM for each group, with group sizes same as in (B). *, **, or *** denote *P*<0.05, *P*<0.01, or *P*<0.001, respectively, in one-way ANOVA applying Tukey's *post-hoc* comparison of all groups using GraphPad Prism 5.0 software. ns, not significant. Asterisks above error bars refer to *post hoc* comparison of *CEACAM1*-humanized mice and wild-type mice exposed to H44/76wt. Asterisks next to bracket refer to *post hoc* comparison of either wild-type or *CEACAM1*-humanized mice (as indicated) with all other groups (infected with H44/76Δ*opa* or heat-inactivated H44/76) at day 55. (F) Meningococcal (H44/76) specific serum IgG concentration at day 55 determined by ELISA in indicated treatment groups of experiment with 10^8^ CFU of the indicated inoculi used for exposures. Group sizes are the same as in (B), right panel. (G) Serum bactericidal antibody (SBA) titres in serum samples taken at day -3 (‘pre’) day 24 or day 55, respectively, of mice exposed to 10^5^ CFU of the indicated inoculi (top panel), or 10^8^ CFU of the indicated inoculi (bottom panel). Each circle represents the result for one individual mouse. Group sizes as well as percentage of SBA-positive mice in each group are indicated in the graph. (H) Correlation of SBA titres as in (G) with meningococcal-specific IgG titres as in (E) for each individual mouse. Where applicable, data were pooled from day 24 and day 55, i.e. up to two data points were obtained from the same mouse. Thresholds indicate minimal IgG concentration above which all samples had positive (≤8) SBA titres. Results of Spearman's rank correlation are shown in each graph.

Exposure to pathogenic bacteria should prompt adaptive immune responses that protect against recurrent infections with the same infectious agent. To assess the adaptive response following colonization, we performed a recurrent infection experiment, in which mice were intranasally infected with 10^5^ CFU of *Nme* H44/76wt, H44/76Δ*opa*, or heat-inactivated H44/76 at the beginning of the experiment (day 0) and again at day 21 (infection schedule depicted in Fig. 6A). Intranasal challenge infections with a high-dose (10^8^ CFU) H44/76 were then performed either at day 21, at which point mice had been previously exposed to a single bacterial inoculation, or at the end of the experiment at day 52, by which point mice had been exposed to the meningococci twice before, and then colonization of the mice was assessed three days post challenge. Unexpected when considering that the mice had been colonized for up to 10 days prior, the *CEACAM1*-humanized mice could be re-infected with the same strain when challenged again on day 21. However, when they were exposed twice before challenge at day 52, meningococcal colonization was almost completely abrogated, indicating a sterilizing immune response (Fig. 6B, left panel). Interestingly, when the mice were exposed twice to 10^5^ viable H44/76Δ*opa* or 10^5^ heat-inactivated H44/76, the former of which can replicate but not adhere to CEACAM1 while the latter can adhere but not replicate, no protection was observed. To address whether this is dose-dependent, we intranasally inoculated mice with 10^8^ H44/76Δ*opa* or heat-inactivated H44/76 before challenging with viable H44/76 to assess colonization (Fig. 6B, right panel). Indeed, in this setting, the mice became protected against colonization with H44/76wt after two exposures, indicating the heightened immune response upon viable wild type *Nme* infection of the transgenic mice results from the increased delivery of antigens as the bacteria replicate in association with the CEACAM1-expressing tissues.

Adaptive mucosal protection against bacteria typically involves IgA that inhibits adhesion and promotes bacterial aggregation, thereby facilitating their removal by cilia movement. Indeed, we found significant levels of *Nme*-specific IgA, but not IgG, in nasal lavage fluids of *CEACAM1*-humanized mice after twice being inoculated with 10^5^ H44/76wt, but not in the other infection groups (Fig. 6C). Protection against colonization correlated significantly with nasal anti-meningococcal IgA levels of individual *CEACAM1*-humanized animals (Fig. 6D, left panel). In *CEACAM1*-humanized mice pre-infected with 10^5^ H44/76Δ*opa* or heat-inactivated H44/76, nasal IgA titers remained low, and did not correlate with colonization (Fig. 6D, middle and right panel). Nasal IgA levels poorly correlated with serum IgA levels and serum IgG levels, suggesting that mucosal IgA is locally produced rather than serum derived (Fig. S6).

Serum *Nme*-specific IgG was found to rise quickly in *CEACAM1*-humanized mice infected with 10^5^ H44/76wt, even before the second intranasal infection (Fig. 6E). In WT mice, a significantly weaker response to H44/76wt emerged only after the second challenge. Exposure to 10^5^ H44/76Δ*opa* or heat-inactivated H44/76 did not elicit any detectable *Nme*-specific IgG. Serum anti-meningococcal IgA levels mirrored the findings for IgG, whereas meningococcal-specific IgM showed only a weak and transient elevation in both, WT and *CEACAM1*-humanized mice (Fig. S7). High concentrations of serum meningococcal-specific IgG were found in mice of both genotypes upon exposure to 10^8^ H44/76Δ*opa* or heat-inactivated H44/76, demonstrating the dose-dependency of this response (Fig. 6F).

The occurrence of *Nme*-specific complement fixing antibodies defines a clinically relevant correlate of protection against invasive disease. The serum bactericidal antibody assay (SBA) was, therefore, used to monitor serum protection of the repeatedly infected mice. Strikingly, about 40% of *CEACAM1*-humanized mice developed positive SBA titres (i.e. ≥8, considering the use of rabbit complement [36]) in response to the first exposure to H44/76 and almost 80% were protected after the second exposure, whereas less than 20% of WT mice showed any SBA titres after the second meningococcal challenge (Fig. 6G, upper panel). Consistent with their low *Nme*-specific Ig titres, mice exposed to 10^5^ H44/76Δ*opa* or heat-inactivated H44/76 did not develop positive SBA titres. However, after high intranasal doses, robust SBA titres did emerge in both these groups (Fig. 6G, lower panel).

Plotting SBA titres versus *Nme*-specific serum IgG of individual animals revealed that persistent growth of Opa-expressing *Nme* in the tissues of *CEACAM1*-humanized mice led to protective SBA titres already at comparatively low titres of *Nme*-specific Ig. The threshold above which all analyzed serum samples were SBA positive (i.e. ≥8) was ∼5,000 ng/ml in *CEACAM1*-humanized mice infected with 10^5^ live H44/76wt (Fig. 6H, upper left panel), compared to ∼15,000 ng/ml in WT mice exposed to the same dose (Fig. 6H, upper right panel). The thresholds of both mouse strains exposed to 10^8^ H44/76Δ*opa* or heat-inactivated H44/76 were in a similar range (13,000–30,000 ng/ml). This suggests that meningococcal growth within the tissues elicits protective immunity more effectively than does a higher dose or repeated exposure to the same antigen.

Interestingly, in *CEACAM1*-humanized mice repeatedly inoculated with Opa-expressing live or heat-inactivated bacteria, there was a very strong and highly significant correlation between serum IgG and SBA titres (Fig. 6H). Therefore, Opa-CEACAM interaction might influence the antibody repertoire or functionality generated in response to intranasal exposure.

### Distinct correlates of protection typify immunity in response to meningococcal colonization and parenteral immunization

**Figure 7:**
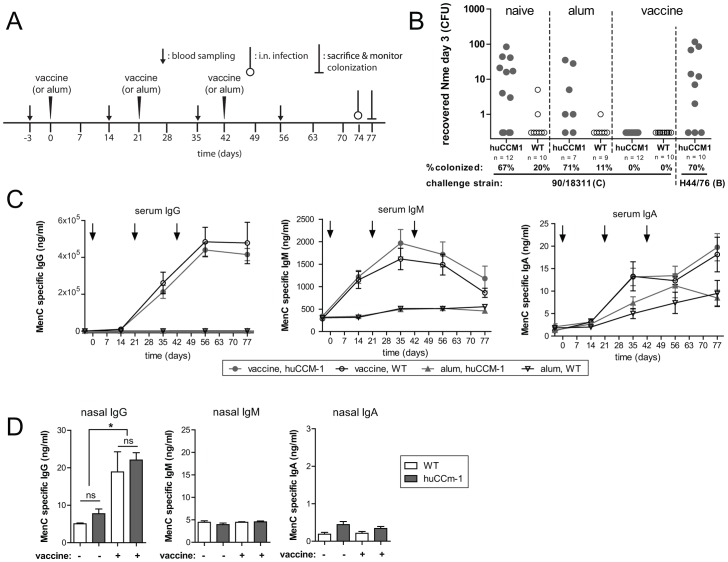
Polysaccharide conjugate vaccine induces sterile immunity in *CEACAM1*-humanized mice. (A) Schematic representation of experiment. Cohorts of mice were vaccinated with serogroup C polysaccharide conjugate vaccine, or alum alone as control, at day 0, 21 and 42 and then i.n. challenged at day 74. (B) Recovered viable CFU at day three after i.n. challenge with 10^8^ CFU of serogroup C strain 90/18311, or serogroup B strain H44/76, as indicated below x-axis. Mouse treatment groups were as follows: ‘naive’ = untreated; ‘alum’ = controls receiving alum alone; ‘vaccine’ = vaccinated mice. (C) *Nme*-specific (strain 90/18311) Ig titres during the course of vaccination. Mean values ± SEM derived from the same groups as in (B) are plotted. Arrows indicate vaccine administration. (D) Meningococcal-specific (strain 90/18311) Ig titres in nasal wash fluid at day 77. Mean values ± SEM derived from the same groups as in (B) are plotted. *, denotes *P*<0.05, in one-way ANOVA applying Tukey's *post-hoc* comparison of all groups using GraphPad Prism 5.0 software. ns, not significant.

A key, yet unexpected, feature governing the success of meningococcal serogroup C polysaccharide vaccines relies on their ability to induce sterilizing immunity within the mucosa [37]. We sought to determine whether the *CEACAM1*-humanized mouse model could serve as platform in which to assess the potential of novel vaccine candidates to protect against nasopharyngeal colonization. Since it represents a ‘gold standard’ as far as efficacy, we sought to test the effect of the serogroup C-conjugated polysaccharide vaccine. First, we established that serogroup C *Nme* could colonize the *CEACAM1*-humanized mice, providing evidence that the dependence on human CEACAM1 was not restricted to serogroups B strains. Next, we immunized the mice with the conjugate vaccine, alum alone, or no vaccine (Fig. 7A and B). The vaccine elicited serogroup C-specific protection, conferring complete protection against the serogroup C strain without affecting infection by serogroup B strain. In both WT and *CEACAM1*-humanized mice, vaccination mounted robust serum *Nme*-specific IgG titers, whereas the rise in *Nme*-specific IgM was transient and only a weak *Nme*-specific IgA response was achieved (Fig. 7C). In contrast to the IgA-dominated mucosal response to meningococcal infection of the *CEACAM1*-humanized mice, immunity conferred by the vaccine correlated with *Nme*-specific IgG without any IgA response being apparent in either mouse genotype (Fig. 7D). Since nasal IgG and serum IgG concentration showed a significant correlation, the nasal IgG appears to be mainly serum derived (Fig. S8).

## Discussion

The intimate relationship between *Nme* and the human host begins with their attachment to the nasopharyngeal mucosa. From here, the bacteria may penetrate into the local submucosa [38] or, in very rare instances, disseminate to cause rapidly progressing invasive disease [39]. Herein, we have used mice expressing human CEACAM1 to establish a colonization model that reveals a central role for human CEACAM1 binding for meningococcal colonization and persistence within the nasopharynx. This development allowed us to consider both bacterial phenotype selection and the relative contribution of immune processes *in vivo*. Consistent with meningococcal infection relying on an ongoing selection for phase variants expressing the phenotype that allows persistence within its niche, bacteria persisting within the nasal passage were uniformly Opa-expressing, even when the mice were inoculated with Opa-negative isolates. This phase variant selection for Opa^+^ variants *in vivo* mirrors previous *in vitro* findings of Opa^+^-selection in primary nasopharyngeal cells [23]. Perhaps more surprisingly, the innate inflammatory response to meningococcal infection relied on bacterial Opa expression and human CEACAM1 expression in the tissues (Fig. 2). In fact, the mucosal inflammatory response in CEACAM1-humanized mouse was the same regardless of whether the Opa-expressing bacteria were alive or dead, while the relatively low cytokine response of these same mice to viable Opa-deficient *Nme* reflected that of Opa-expressing bacteria in wild-type mice (Fig. 4). The efficient CEACAM1-dependent engulfment (Fig. 1B) and/or delivery of Opa-expressing bacteria to the submucosal tissues (Fig. 4B) presumably explains this effect.

Since this work focused on the functional interplay between meningococcal Opa proteins with human CEACAM1, we cannot exclude any contribution of other factors, such as the pilus. While the pilus cannot interact with murine cells, it promotes interbacterial tethering that culminates in the formation of microcolonies [40]. However, we observed successful colonization with the serogroup C strain 90/18311 (Fig. 7B) which is not piliated (Fig. S1A) and - unlike that seen with Opa proteins (Fig. 2A) - there was no selection during colonization for pilus-expressing phenotypes (data not shown).

While disease caused by *Nme* occurs when these bacteria proliferate systemically, the absence of a bacteremic outcome in our model reflects normal colonization in humans. In fact, considering that less than 1 in 25,000 natural infections in humans lead to invasive meningococcal disease during endemic periods [41], it seems likely that some as yet unidentified genetic and/or environmental cofactor(s) contribute to disease. Various groups have used neonatal and/or iron supplementation of mice to study invasive disease [5], [6], however these do not consider mucosal colonization. One study did establish persistent infection after intranasal inoculation of Swiss-Webster mice, however there is no basis to compare our studies since they did not localize the bacteria within the tissues [7] and we did not see any persistence of three different strains in the wild type FvB littermates of our CEACAM1 transgenic animals. The introduction of human alleles encoding other proteins targeted by other meningococcal virulence factors has proven fruitful for understanding invasive disease. Transgenic mice expressing the human complement regulator membrane protein, CD46, reported to bind the neisserial pilus, are more susceptible to disseminated meningococcal infection [4], [35]. One particularly important advance has been the demonstration that transgenic mice expressing the human serum iron transport protein transferrin are susceptible to invasive meningococcal disease because *Neisseria sp*. can readily access this iron pool [3], [8]. Combining the CEACAM1 colonization model with these transgenes and/or environmental insults such as viral co-infection [3], [42], smoking [43] or extremes in humidity [41] may ultimately prove informative to understanding the transition from asymptomatic infection to disseminated disease.

Individuals with complement deficiency have heightened susceptibility to invasive meningococcal disease [44], prompting us to explore whether this key innate defense affected meningococcal colonization of the nasopharynx. It has been speculated that capsule is not necessary for nasopharyngeal colonization, since about 16% of meningococci isolated from healthy carriers are devoid of the capsule operon [29]. Three different capsule-deficient *Nme* strains used in this study colonized *CEACAM1*-humanized mice successfully at different frequencies (23%, 30% and 100%, respectively; Fig. 5A), reflecting the situation in humans well. The finding that loss of capsular expression had a more marked effect on MC58 than it did on H44/76 was unexpected. Considering that these strains are both typed as B:15:P1.7,16 and ST-32, and that genome alignments reveal a high degree of sequence similarity between them [38], [39], it seems most plausible that the phase variation and/or antigenic variability of some as yet uncharacterized virulence factor may account for this difference. Still, most meningococci in human carriers are encapsulated, and our model suggests that the capsule can facilitate their survival within the mucosa.

Global activation of the complement cascade using cobra venom factor conferred protection against subsequent meningococcal challenge, suggesting that the meningococci's ability to bind complement-regulatory factors [45], [46], [47], [48], [49] may contribute to its fitness within the mucosa rather than just during invasive disease. Perhaps surprisingly, however, mice that were depleted of late complement components were not more susceptible to meningococcal colonization unless neutrophils were simultaneously depleted (Fig. 5C), implying a buttressed defense with both factors contributing to protection.

The absence of a relevant experimental model in which to assess colonization has led to serum bactericidal antibody becoming the primary correlate of protection for any meningococcal vaccine candidate. Our observation that the SBA titres did not strictly correlate with sterilizing immunity (Fig. 6B and 6G) is, therefore, both unexpected and enlightening. This presumably results, at least in part, from the requirement for *Nme*-specific Ig at the mucosal surface to confer protection (Fig. 6C). Notably, while heat-inactivated *Nme* elicited a cytokine response indistinguishable from that towards viable Opa-expressing meningococci, *Nme*-specific IgA did not arise unless viable bacteria persisted in the tissues. Since herd immunity relies on the eradication of *Nme* carriage, this has obvious implications for the advent of any nasal-targeted vaccine.

In contrast to naturally-acquired immunity, the serogroup C capsule-conjugate vaccine generated *Nme*-specific IgG in the mucosa without any IgA response (Fig. 7). It is important to consider that the conjugate vaccine-induced response targets the serogroup C capsule, whereas the sialic acid-based serogroup B capsule is non-immunogenic, implying that the differences in immune response may reflect this difference in antigen composition. However, our findings mirror observations made in human vaccinees that received serogroup C meningococcal vaccines, in which memory responses yielded nasal IgG but not IgA [37], [50]. While the human studies could not challenge these individuals with *Nme*, our studies confirm that the systemic IgG response arising from parenteral immunization penetrates the mucosa so as to confer sterilizing immunity. Parenteral immunization thus tends to elicit a systemic response that promotes SBA and mucosal immunity, whereas nasal infection can produce localized protection with little systemic Ig. When considering these differences, they must make us pause regarding the strict reliance on SBAs when considering the potential susceptibility of individuals either prior to or post-vaccination with meningococcal vaccines.

## Materials and Methods

### Bacterial strains

*N. meningitidis* strains were grown on GC agar (Becton Dickinson, Sparks, USA) supplemented with IsoVitalex (Becton Dickinson, Sparks, USA) at 37°C with 5% CO_2_ and a water saturated atmosphere. Details about strains and their mutants are reviewed in table S1. Opa and pilin expression status, confirmation of capsule type (and absence in knockout mutants) and comparative growth curves of mutants and parental strains of all meningococci used in this study were determined by western blotting as shown in Fig. S1.

For expression in *E.coli*, all four different *opa* genes from *N. meningitidis* MC58 were subcloned into pTrc99A vector. *E. coli* were grown on LB agar at 37°C. To induce expression in transformants of the *opa* genes, IPTG was added to the growth media at 40 µg/ml.

### Cell lines and culture conditions

All cells were incubated in 37°C incubators equipped with 5% CO_2_ and a water-saturated atmosphere. Hela cells stably expressing CEACAMs or the empty vector were maintained in RPMI+10%FBS. Primary normal nasal epithelial cells HNEPc were purchased from Promocell (C-126200) and cultured in Keratinocyte serum-free media supplemented with 0.05 mg/ml bovine pituitary extract and 5 ng/ml epidermal growth factor (Gibco 17005-057).

### Gentamycin protection assay

Binding of *E. coli* expressing *N. meningitidis* MC58 Opa proteins and their invasion into human *CEACAM1*, *CEACAM3*, *CEACAM5* or *CEACAM6* expressing HeLa cells was performed as described elsewhere [9].

### Western blot detecting Opa and pilin

Bacterial suspensions of about 10^10^/ml were admixed with an equal volume of twofold concentrated SDS loading buffer containing 10% beta-mercaptoethanol and were boiled for 10 min prior to analysis on 12% (for detection of Opa protein) or 15% (for detection of pilin) SDS-polyacrylamide gel electrophoresis and subsequent transferred to nitrocellulose membrane (Hybond C-extra, Amersham Biosciences, Little Chalfont, UK). Opa proteins were detected using mouse monoclonal antibody 4B12C11, which detects most gonococcal and meningococcal Opa proteins [51]. Pilin was detected using mouse monoclonal antibody 10H5.1.1, a kind gift of Dr. Maggie So (University of Arizona, Tucson, USA).

### Mouse strains

Generation of CEACAM1-humanized mouse line was described in Gu *et al.* [20] and CEABAC mice expressing human CEACAM3, 5, 6, 7 were described by Chan and Stanners [52]. Both mouse lines were on FvB background. In each case, transgenic animals were bred with wild-type animals to provide wild type littermates as controls.

### Mouse intranasal infection

All animal experiment procedures approved by the Animal Ethics Review Committee of the University of Toronto (Permit Numbers: 20008007 and 20008657), which is subject to the ethical and legal requirements under the province of Ontario's Animals for Research Act and the federal Council on Animal Care (CCAC). All efforts were made to minimize suffering. The infection protocol used in this study is similar as described in Yi *et al.* [7]. For intranasal infection, six week old mice were anesthetized with Isofluran (Baxter, Missisauga, Canada) inhalation and a total of 10 µl inoculum containing the indicated amount of meningococci were applied to both nares. An overnight lawn of growth of meningococci was harvested into 1 ml of PBS containing 1 mM of MgCl_2_ (PBS/Mg) and OD600 was measured to adjust the number of bacteria. For inoculum preparation, one volume of bacterial suspension was mixed with one volume of sterile filtered 32 mg/ml human holo-transferrin in PBS/Mg (Sigma Aldrich, Oakville, Canada). To ensure bacterial dosage in every experiment, serial dilutions were plated onto GC agar supplemented with IsoVitalex. At indicated time points, animals were sacrifized by CO_2_ inhalation and blood was drawn by cardiac puncture. CFU counts were assessed by retrograde lavage of the upper airways through the trachea with 0.5 ml of PBS/Mg and swabbing of the exposed nasal cavities using aluminum shaft applicators (Puritan Medical Products, Guilford, USA) resuspended into 500 µl of PBS/Mg. Serial dilutions of samples were plated onto GC agar plates supplemented with IsoVitalex and VCNT inhibitor (Becton Dickinson, Sparks, USA) to suppress outgrowth of nasal flora. After overnight incubation of inoculated plates, meningococcal colonies were enumerated and expressed as the sum of recovered colony forming units (CFU) from each mouse.

### Mouse intraperitoneal infection

For intraperitoneal injection, the overnight growth of meningococci from a GC agar plate supplemented with IsoVitalex was resuspended in 10 ml of Brain-Heart- Infusion (BHI) (Becton Dickinson, Sparks, USA) supplemented with 60 µg/ml Deferoxamine mesylate (Sigma Aldrich, Oakville, Canada) as iron chelator and incubated at 37°C under constant agitation for 4 h. Then, the OD_600_ of the suspension was assessed and the inoculum adjusted in BHI. Serial dilutions of the inoculum were plated onto GC agar plates supplemented with IsoVitalex to ensure correct bacterial concentration. Six to eight week old mice were injected 200 µl of inoculum and, at a different site, 200 µl sterile saline containing iron dextran (Sigma-Aldrich, St. Louis, USA) commensurate with 2 mg Fe^3+^ as a source of iron.

### Mouse vaccination

For immunization, the meningococcal serogroup C polysaccharide conjugate vaccine NeisVac-C (Baxter, Mississauga, Canada) was used. Mice received 1 µg of polysaccharide, conjugated to 1–2 µg of tetanus toxoid and adsorbed to 50 µg alum, corresponding to 1/10 of a single dose as purchased from manufacturer, subcutaneously on day 0, 21 and 42. Control groups received an equal amount of alum (alhydrogel, Invivogen, San Diego, USA) alone instead.

### *In vivo* depletion of complement and neutrophils

Where indicated, mice were injected intraperitoneally with 200 µl sterile saline containing 20 µg with Cobra Venom Factor (CVF) (Quidel, San Diego, USA) at either 4 h or 30 h prior to infection to either over-activate the complement system or completely deplete the mice of serum complement, respectively.

Neutrophil depletion for up to three days was achieved by a single i.p. injection of 200 µl of sterile saline containing 250 µg of RB6-8C5 (hybridoma line courtesy of Prof. Paul Allen, Department of Pathology and Immunology, Washington University School of Medicine, St. Louis) at 24 h prior to infection. If neutrophil depletion was needed for a longer period of time, the RB6-8C5 injection was repeated every 48 h.

### Western blot detecting mouse complement C3

Five µl of tail vein blood were obtained from mice and immediately diluted in 45 µl of ice-cold PBS containing 10 mM EDTA to inhibit complement degradation. Cellular components were removed by brief centrifugation at 1000× g for 1 min and supernatant was diluted 1∶10 in PBS, 10 mM EDTA. An equal volume of 2× SDS buffer without reducing agent was added and the sample was not boiled prior to loading onto 6% SDS-PAGE to avoid dissociation of C3 into its alpha and beta chain. After transfer to nitrocellulose membrane (Hybond C-extra, Amersham Biosciences, Little Chalfont, UK), mouse C3 was detected using goat-anti-mouse C3 antiserum (Genetex, Irvine, CA, USA).

### Immunohistochemistry

Paraffin-embedded skull sections were stained for human CEACAM1 using CEA-Dako or a matched negative control (Dako, Burlington, Canada). Mouse Ceacam1 was detected using rabbit-anti-mouse Ceacam1 (generous gift from Prof. Nicole Beauchemin, McGill University, Montreal) or normal rabbit serum as negative control. For detection of neutrophils, NIMP-R14 or a rat IgG isotype control (Abcam, Cambridge, USA) was used and the signal amplified with goat-peroxidase-anti-peroxidase conjugate (Jackson Immunoresearch, West Grove, USA). HRP-conjugated secondary antibodies were obtained from Jackson Immunoresearch, West Grove, USA. Visualization was achieved by incubation with 3,3′-Diaminobenzidine (Sigma-Aldrich, Oakville, Canada) according to manufacturer's recommendations. After immunostaining, nuclei were counterstained using Harris' Hematoxylin (VWR, West Chester, USA) and samples were dehydrated in an ascending ethanol/xylene series and mounted with SHUR/Mount (Triangle Biomedical Sciences, Durham, USA).

### Immunofluorescence microscopy

Paraffin-embedded sections of mouse skulls were stained for human CEACAM using mouse monoclonal D14HD11 (Abcam, Cambridge, USA) and meningococci were detected by rabbit polyclonal anti-meningococcal antiserum. Secondary antibodies were goat-anti-mouse-IgG-Alexa647 and goat-anti-rabbit-IgG-Alexa594 (Life Technologies, Burlington, Canada). Autofluorescence was quenched by 10 min incubation in 0.3% Sudan black (Sigma-Aldrich, Oakville, Canada) in 70% ethanol, samples washed with PBS and mounted with Prolong Gold antifade with DAPI (Life Technologies, Burlington, Canada). Images were taken on a Leica DM IBRE epifluorescence microscope (Leica, Wetzlar, Germany).

### *In vitro* association of *N. meningitidis* by primary murine neutrophils

Primary murine neutrophils were isolated from bone marrow of femur and tibia of CO_2_ euthanized mice and purified on a discontinuous Percoll gradient (80%/65%/55%). PMNs were recovered at the interface between the 80% and 65% Percoll solution and washed in PBS. Purity of neutrophils using this technique is usually greater than 90%. PMNs were seeded onto mouse-serum coated coverslips at a density of 5×10^5^ in 500 µl in DMEM supplemented with 5% FBS, spun down for 10 min at 63× g and let rest for 3 h at 37°C, 5% CO_2_. Then, cells were infected at a multiplicity of infection of approximately 25 of freshly harvested meningococci grown overnight on GC agar plates supplemented with IsoVitalex. The bacteria were spun onto the PMNs by centrifugation at 63× g for 5 min and incubated at 37°C, 5% CO_2_ for 30 min. Then, samples were washed with HBSS and fixed with 3.7% paraformaldehyde. *Nme* were stained using rabbit polyclonal anti-meningococcal antiserum, actin as stained using alexa 488-labeled phalloidin and nuclei were stained using DAPI. Twenty-five to fifty cells per condition were randomly imaged and the number of adherent/internalized bacteria manually quantified.

### Enzyme-linked immunosorbent assay (ELISA) for detection of cytokines

Nasal wash fluid was collected from mice as described in the intranasal infection procedure, but 0.7 ml PBS containing 5 mM EDTA, 2 µg/ml Aprotinin, 2.5 µg/ml Leupeptin and 1 µg/ml pepstatin (all obtained from Sigma-Aldrich, Oakville, Canada) were used for lavage. When nasal swab sample were collected, they were resuspended into the nasal wash fluid obtained from the same mouse (i.e. the nasal tissue sample for each mouse contained nasal wash and mucosal tissue and debris collected with the swab). The samples were kept on ice until further processing. The samples were spun on a tabletop centrifuge at 13,000 rpm for 10 min at 4°C and the supernatant recovered and sterile filtered through 0.22 µm cellulose acetate SpinX columns (Corning Inc., Corning, USA). Samples were used neat or in 1∶10 dilution for chemokine ELISAs using DuoSet ELISA Development System kits for mouse CXCL-1/KC, CCL3/MIP-1α or mouse CXCL-2/MIP-2 (R&D Systems, Minneapolis, USA), and for cytokine ELISAs using BD OptEIA mouse TNF ELISA Set II and mouse IL-1β ELISA set (BD Biosciences Pharmingen, San Diego, USA), according to the manufacturer's instructions.

### Myeloperoxidase assay

Pellet of cells and tissue debris from mouse nasal samples after centrifugation described above for cytokine ELISAs was used to determine myeloperoxidase activity as marker for neutrophil infiltration. Pellets were resuspended in 300 µl of 50 mM potassium phosphate buffer pH 6,0 containing 50 mM Hexadecyltrimethylammonium bromide (HTAB) and homogenized for 10 s. Then, 700 µl of 50 mM potassium phosphate buffer pH 6,0 were added and sonicated for 30 s. The samples were snap-frozen and thawed three times and centrifuged 10 min at 13,000 rpm on a tabletop centrifuge and supernatants were transferred to Spinx columns for sterile filtration as above. Peroxidase activity was measured relative to a standard curve prepared from human myeloperoxidase (Sigma-Aldrich, Oakville, Canada) by incubation with SureBlue peroxidase substrate (KPL, Gaithersburg, USA), following manufacturer's instructions.

### Meningococcal-specific Immunoglobulin ELISAs

Maxisorp 96 well flat-bottom immuno plates (Nunc, Rochester, USA) were coated with 50 µl per well of a suspension at OD_600_ of 0.2 of heat-inactivated (56°C for 30 min) *N. meningitidis* H44/76 in PBS and allowed to dry. Wells were washed four times with wash buffer (PBS containing 0,05% Tween-20) and blocked 1 h with PBS containing 5% BSA before addition of 50 µl per well of diluted sample. After incubation for 2 h at room temperature, wells were washed three times and 50 µl per well of 1∶10,000 dilution of AP-goat-anti-mouse IgG Fc(γ) or AP-goat-anti-mouse IgM (Jackson Immunoresearch, West Grove, USA), or AP-goat-anti-mouse IgA (Abcam, Cambridge, USA), were added. After 1 h incubation, wells were washed thrice and 100 µl per well of BLUEPHOS AP detection substrate (KPL, Gaithersburg, USA) were added and plates incubated at 37°C. Then, the reaction was stopped by adding 100 µl/well of AP-Stop solution (KPL, Gaithersburg, USA) and OD_620_ was measured.

### Serum bactericidal assay (SBA)

Complement-mediated serum bactericidal antibody activity was measured using washed, exponential growth-phase bacteria grown to OD_600_ of 0.6 in BHI supplemented with 0.25% glucose and 0.02 mM CMP-NANA. For measuring bactericidal activity, the mouse sera were heat-inactivated (56°C for 30 min) to remove endogenous complement activity and were added to the bacteria in serial dilutions. Baby rabbit complement (Pel-Freez, Rogers, USA) was added as exogenous source of complement at a final concentration of 20%. An aliquot of each reaction was plated onto GC agar plates supplemented with IsoVitalex directly upon addition of the complement (t = 0) and after 1 h incubation of the mixture at 37°C. Viable CFU were enumerated and serum bactericidal antibody assay for each mouse serum dilution considered positive when CFU counts after 1 h incubation were <50% of those at t = 0.

## Supporting Information

Figure S1 **Phenotyping of meningococcal strains used in this study.** (A) Western blot analysis detecting Opa protein expression (top) and pilin expression (middle) as well as dot-blot of washed intact, heat-inactivated bacteria spotted onto nitrocellulose membrane probing for piliation (bottom). All strains except serogroup C strain 90/18311 were piliated. (b) Dot-blot of washed intact, heat-inactivated bacteria spotted onto nitrocellulose membrane probed with serogroup B (top) or serogroup C (bottom) specific antibodies. MC58Δ*siaD* and H44/76Δ*siaD* mutants were confirmed to be non-capsulated. (C) Confirmation of Opa protein expression in *E. coli* mutants transformed with constructs encoding OpaA, J, B, D or *Nme* strain MC58. (D) Growth curves of MC58 (top panel) and H44/76 (bottom panel) and their mutants, respectively, obtained in BHI broth. No obvious differences were found between mutants and corresponding parental strains. (E) Silver-stained SDS gels analyzing overall protein expression pattern of MC58, MC58Δ*siaD*, H44/76 and H44/76Δ*opa*. No obvious differences were found between mutants and parental strains.
(EPS)

Figure S2 **CEACAM1/Ceacam1 expression in polymorphonuclear cells and efficiency of neutrophil depletion.** (A) Adjacent sections from the same specimens as in Fig. 4A were stained for human CEACAM (top panels), or mouse Ceacam1 (middle panels), or with an isotype control (bottom panels) and counterstained with Hematoxylin. Same regions of interest as in Fig. 4A were chosen from microscopy at 40× magnification. Insets show enlargements of PMN infiltrates for better visualization. (B) Demonstration of epithelial damage in response to infection with *Nme* after neutrophil depletion. Sections as above (groups in same sequence) were stained with Gr-1 antibody detecting PMNs and counterstained with Hematoxylin. Microscopic images shown were obtained at 40× magnification. Marked lesions of epithelia are indicated by arrows. (C) Mice were i.p. injected with the indicated amounts of anti-Gr-1 antibody clone RB6-8C5 and at indicated time points, blood smears were obtained for enumeration of cells. Presented is the fraction of PMNs relative to all nucleated cells in the sample. (D) Prior to intranasal infection with 10^8^ CFU of MC58, mice were either left untreated, or injected with 250 µg of RB6-8C5 once only, or injected with 250 µg of RB6-8C5 before and then every 48 h after infection, and blood samples were taken for enumeration of PMNs as in (C).
(EPS)

Figure S3 ***In vitro*** **association of** ***N. meningitidis*** **with bone-marrow derived mouse neutrophils.** (A) Immunofluorescence images of primary neutrophils from WT mice or *CEACAM1*-humanized mice infected with H44/76 or H44/76Δ*opa* for 30 min at an MOI of approximately 25. Bacteria appear in red, actin in green and nuclei in blue. Three different regions of interest are depicted for each condition. (B) Quantification of cell-associated bacteria at 30 min post infection as in (A) was manually determined from 25–50 randomly chosen regions of interest. Depicted are the results from three independent experiments, each plotting the mean number of bacteria per cell ± SEM for each group.
(EPS)

Figure S4 **Selection for Opa^+^ variants during intranasal colonization of PMN-depleted mice.** (A) PMN-depleted mice were i.n. infected with 10^8^ CFU of MC58, in which about half of the bacteria had turned off Opa expression by phase variation. At day 1 or day 3, mice were sacrifized and viable meningococci recovered from nasal tissues for quantitative culture. Each circle represents number of CFU recovered from one mouse. (B) Colonies were randomly chosen from plated inoculum (i), or from plates inoculated with samples from WT and *CEACAM1*-humanized mice at day 1 (ii), or day 3 (iii), immobilized onto immunosorbent 96-well-plates and probed for binding of soluble CEACAM1. (iv) Western blot showing that CEACAM1-binding clones expressed Opa proteins while non-binders did not. (C) Fraction of CEACAM1-binding colonies/total colonies as assayed in (B). Numbers above bars indicate number of CEACAM1-binding colonies/total numbers of colonies analyzed in the corresponding group. * or **** denote *P*<0.05 or *P*<0.0001, respectively, applying Fisher's exact test using GraphPad Prism 5.0 software.
(EPS)

Figure S5 **Efficiency of Cobra Venom Factor (CVF) to deplete complement.** (A) A cohort of 8 mice was i.p. injected with 20 µg of CVF and the control group of 6 mice was left untreated. At day 3, plasma samples were taken and analyzed on a 6% non-reducing SDS-PAGE followed by Western blot probing for complement component C3 (∼180 kDa). (B) Four mice received 20 µg CVF by i.p. injection and plasma samples were obtained at indicated time points for Western blot detection of complement component C3 as in (A).
(EPS)

Figure S6 **Correlation of** ***Nme*** **-specific nasal IgA with serum** ***Nme*** **-specific IgA and IgG.** Data are the same as in Fig. 6C (nasal IgA concentration), Fig. 6E (serum IgG concentration) and Fig. S7 (serum IgA concentration), from *CEACAM1*-humanized mouse cohort repeatedly infected with strain H44/76wt. Results from Pearson's correlation are indicated in each graph.
(EPS)

Figure S7 **Serum anti-** ***Nme*** **IgA and IgM after intranasal exposure to** ***N. meningitidis*** **.** (A) Meningococcal-specific serum IgA and (B) meningococcal-specific IgM concentration was determined by ELISA in indicated treatment groups exposed to 10^5^ CFU of indicated inoculi (same experiment as in Fig. 6). Represented are the mean values ± SEM for each group with groups identical to those in Fig. 6E. *, **, or *** denote *P*<0.05, *P*<0.01, or *P*<0.001, respectively, in one-way ANOVA applying Tukey's *post-hoc* comparison of all groups using GraphPad Prism 5.0 software. ns, not significant. Asterisks above error bars refer to *post hoc* comparison of *CEACAM1*-humanized mice and WT mice exposed to H44/76wt. Asterisks next to bracket refer to *post hoc* comparison of either WT or *CEACAM1*-humanized mice (as indicated) infected with H44/76wt with all other groups (infected with H44/76Δ*opa* or heat-inactivated H44/76) at day 55.
(EPS)

Figure S8 **Correlation of** ***Nme*** **-specific nasal IgG with serum** ***Nme*** **-specific IgG.** Data are the same as in Fig. 7C (serum IgG concentration) and Fig. 7D (nasal IgG concentration), from *CEACAM1*-humanized mouse cohort immunized with MenC polysaccharide conjugate vaccine. Results from Pearson's correlation performed using GraphPad Prism 5.0 software are indicated in graph.
(EPS)

Table S1 ***Neisseria meningitidis*** **strains and mutants.**
(EPS)
